# Linc01234 promotes cell proliferation and metastasis in oral squamous cell carcinoma via miR-433/PAK4 axis

**DOI:** 10.1186/s12885-020-6541-0

**Published:** 2020-02-10

**Authors:** Deyu Liu, Xinchun Jian, Pu Xu, Rong Zhu, Yuan Wang

**Affiliations:** 1 Department of Oral and Maxillofacial Surgery, Affiliated Haikou Hospital, Xiangya Medical College, Central South University, Haikou, 570208 China; 2 Department of Oral and Maxillofacial Surgery, Xiangya Hospital, Central South University, Changsha, 410008 China; 3 Cancer Research Institute, Central South University, Changsha, 410008 China

**Keywords:** OSCC, Linc01234, miR-433, PAK4, ceRNA

## Abstract

**Background:**

Increasing studies have demonstrated that long non-coding RNAs (lncRNAs) play an important role in tumor progression. However, the potential biological functions and clinical importance of Linc01234 in oral squamous cell carcinoma (OSCC) remain unclear.

**Methods:**

We evaluated the expression profile and prognostic value of Linc01234 in OSCC tissues by RT-qPCR. Then, functional in vitro experiments were performed to investigate the effects of Linc01234 on tumor growth, migration and invasion in OSCC. Mechanistically, RT-qPCR, bioinformatic analysis and dual luciferase reporter assays were performed to identify a competitive endogenous RNA (ceRNA) mechanism involving Linc01234, miR-433-3p and PAK4.

**Results:**

We found that Linc01234 was clearly upregulated in OSCC tissues and cell lines, and its level was positively associated with T stage, lymph node metastasis, differentiation and poor prognosis of patients with OSCC. Our results shown that Linc01234 inhibited cell proliferation and metastatic abilities in CAL27 and SCC25 cells following its knockdown. Mechanistic analysis indicated that Linc01234 may act as a ceRNA (competing endogenous RNA) of miR-433-3p to relieve the repressive effect of miR-433-3p on its target PAK4.

**Conclusions:**

Our results indicated that Linc01234 promotes OSCC progression through the Linc01234/miR-433/PAK4 axis and might be a potential therapeutic target for OSCC.

## Background

Oral squamous cell carcinoma (OSCC) is one of the most prevalent subsets of head and neck cancers, ranking as the eighth most common cancer among all malignant tumors worldwide [1]. A new global cancer statistic in 2018 reported that 447,751 newly diagnosed cases of oral cancer and oropharyngeal tumors worldwide [2]. Although new technologies in diagnosis and treatment have greatly improved the overall survival rates, the prognosis of OSCC still remains dismal [3]. Therefore, it’s necessary to explore the underlying mechanism and provide novel therapeutic targets for OSCC.

Recently, it has been verified that long noncoding RNAs (lncRNAs) function as oncogenes or tumor suppressors to regulate the biological behaviors of diverse neoplasms [4, 5]. At present, numerous studies have confirmed that lncRNAs are involved in cell proliferation, apoptosis, migration and metabolism in OSCC [6]. For example, lncRNA LEF1-AS1 is remarkedly upregulated in OSCC tissues and servs as an oncogene in OSCC by suppressing the Hippo signaling pathway [7]. LncRNA MEG3 exerted an antitumor effect on cell growth and metastasis in OSCC by suppressing the activity of the WNT/β-catenin pathway [8]. In addition, aberrant lncRNAs also serve as prognostic indicators for tumor recurrence and metastasis. For example, SNHG15 was reported to be significantly upregulated in tumors and enhanced SNHG15 expression could be a promising biomarker for cancer diagnosis, prognosis or treatment [9]. However, the detailed biological importance of most lncRNAs in OSCC development remains unclear.

Linc01234 (ENSG00000249550) is a conserved lncRNA located at 12q24.13 that is aberrantly expressed in several cancers [10–12]. Linc01234 was closely correlated with poor survival in OSCC and breast cancer through analyses of the TCGA database [13, 14]. Although Linc01234 has been reported as an oncogene promoting OSCC growth and inhibiting apoptosis [11], the clinical importance and underlying mechanism of Linc01234 in OSCC progression is still unclear.

Here, our study was conducted to examine the expression levels of Linc01234 in clinical OSCC samples and a series of OSCC cell lines. Then, we investigated the effects of Linc01234 on cell proliferation and migration in OSCC through gain-of-function and loss-of-function experiments. The present study also analyzed the association between Linc01234 expression and the clinical features and prognosis of OSCC patients and might provide new light on targeted therapy and the diagnosis of oral cancer.

## Methods

### Clinical specimens of OSCC

Eighty-eight pairs of OSCC samples were randomly collected from OSCC patients who underwent surgery at the Affiliated Haikou Hospital and Xiangya Hospital, Central South University from 2012 to 2014. None of OSCC patients received radio- or chemotherapy before their surgery. All tumor tissues and their adjacent noncancerous tissues were immediately frozen stored in liquid nitrogen for subsequent RNA extraction. This study was approved by the Ethics Committee of Affiliated Haikou Hospital and Xiangya Hospital, and all OSCC patients provided the informed consent.

### Cell culture

The CAL27(ATCC® CRL-2095), SCC9(ATCC® CRL-1629) and SCC25(ATCC® CRL-1628) cell lines were obtained from American Type Culture Collection (Manassas, VA, USA), The HSC3, NOK and CAL33 cell lines were kindly gifted from Guanghua School of Stomatology of Sun yet-san University. CAL27, CAL33 and HSC3 cells were cultured in DMEM (Gibco, NY, USA) supplemented with 10% FBS (Invitrogen, Carlsbad, CA, USA). SCC25 and SCC9 cells were cultured in DMEM/F-12 (Gibco, NY, USA) supplemented with 10% FBS. Normal oral keratinocytes (NOKs) were cultured in KSFM (Gibco, NY, USA) supplemented with EGF. All cell lines were cultured in a 37 °C, 5% CO_2_ incubator (Additional file 4: Figure S4).

### Cell transfection

Linc01234 siRNAs and control siRNAs were purchased from RiboBio (Guangzhou, China). CAL27 and SCC25 cells were seeded in the 6-well plate and added with 5 μL of siRNAs or siNCs (50 nM) and 5 μL of Lipofectamine 2000 (Invitrogen, Carlsbad, USA) in each well following the manufacturers’ instructions.

### Subcellular fraction and real-time quantitative PCR (RT-qPCR)

The PARIS Kit purchased from Invitrogen (Carlsbad, CA, USA) was applied to isolate cytoplasmic and nuclear RNAs using a previously established protocol, followed by RT-qPCR detection [15]. Total RNA from OSCC tissues and cells was collected using the TRIzol Reagent (Invitrogen Life Technologies) and was subsequently reverse transcribed to cDNA using the PrimeScript RT reagent Kit (Takara, Tokyo, Japan). RT-qPCR detection was performed on a Roche LightCycler 480 system (Bio-Rad, Hercules, CA, USA) using a SYBR Green qPCR Mix (Takara). The relative RNA expression was calculated using the (2^−ΔΔCt^) method. The ΔCt values were normalized to these of GAPDH or U6.

### EdU assay

After 48 h transfection, CAL27 and SCC25 cells (2 × 10^4^/well) were seeded in 24-well plates. The 5-ethynyl-2′-deoxyuridine (EdU) assay (Life Technologies Corporation, USA) was used to evaluate the proliferation ability of OSCC cells as previously reported [16]. Briefly, CAL27 and SCC25 cells were incubated with 100 μL EdU reagent for 2 h at 37 °C, and stained with DAPI and visualized by a fluorescence microscope (Olympus, Tokyo, Japan).

### Cell counting Kit-8 (CCK-8) assay

CAL27 and SCC25 cells were cultured in 96-well plates at 8000cells/well after transfection. Following the 4 consecutive days culture, each well was replaced by the fresh medium containing 10% CCK-8 solution (Yeasen, Shanghai, China). After a 2 h incubation at 37 °C, the absorbance of 450 nm was measured using a microplate reader (Bio-Rad, Hercules, CA, USA).

### Transwell assays

The migration and invasion ability were assessed using Transwell chamber with 8 μm pore (Corning, New York, NY, USA). To evaluate the invasion capacity, OSCC cells (1 × 10^5^) suspended in serum-free DMEM were added into the upper chamber precoated with Matrigel matrix (BD Biosciences, San Jose, CA, USA). For migration assay, OSCC cells (1 × 10^5^) were cultured in the Boyden chamber without Matrigel. After 24 h incubation, the migrated and invaded cells were fixed and stained, and counted under a microscope (Leica, Wetzlar, Germany).

### Wound-healing assays

CAL27 and SCC25 cells were seeded in the 6-well plate and transfected with siRNAs or siNCs. Until a 90% confluence, we generated a scratch on the bottom of each well. After washing with PBS, cells were photographed using a microscope (Leica, Wetzlar, Germany) at 0 h and 48 h.

### Dual-luciferase reporter assays

The sequence of Linc01234 or PAK4 3′-UTR containing the putative or mutated binding sites for miR-433-3p were cloned into the pMIR-REPORT vector (Promega, Madison, WI, USA). The wild-type or mutant pMIR-REPORT vectors were co-transfected into CAL27 and SCC25 cells as long with miR-433-3p mimics and miR-NC. Fourty-eight hour later, the relative luciferase activity was assessed using a dual luciferase assay kit (Promega) and these values were normalized to Renilla activity.

### Western blot assays

Total protein from CAL27 and SCC25 cells was lysed in RIPA buffer (Beyotime, China). Then, the lysates were treated with a 10% SDS-PAGE gel and transferred onto PVDF membranes (Millipore Corporation, USA). After 1 h incubation in 5% nonfat milk solution, the PVDF membranes were cultured with anti-PAK4 (ab62509, Abcam, Cambridge, UK) and anti-GAPDH (AC003, ABclonal, China) antibodies overnight at 4 °C. After TBST washing, the membranes were incubated with the matched secondary antibodies (Proteintech, wuhan, China) at 37 °C for 1 h. The reaction was visualized by an enhanced chemiluminescence (ECL) detection system (Millipore, MA, USA).

### Statistical analysis

All data in this study were performed with SPSS 22.0 (IBM Corp., Armonk, NY, USA) and were expressed as the mean ± standard deviation of at least three independent experiments. CCK8 experiments, colony formation assay, Transwell, wound-healing and dual-luciferase reporter assays, RT-qPCR and Western blotting, were each independently repeated 3 times. Comparisons were performed using two-tailed Student’s *t*-test or one-way ANOVA. The correlation between Linc01234 expression and clinicopathological parameters was analyzed using the χ2 test. *P* < 0.05 was considered to indicate a statistically significant difference.

## Results

### Linc01234 is increased in OSCC tissues and cell lines

**Fig. 1:**
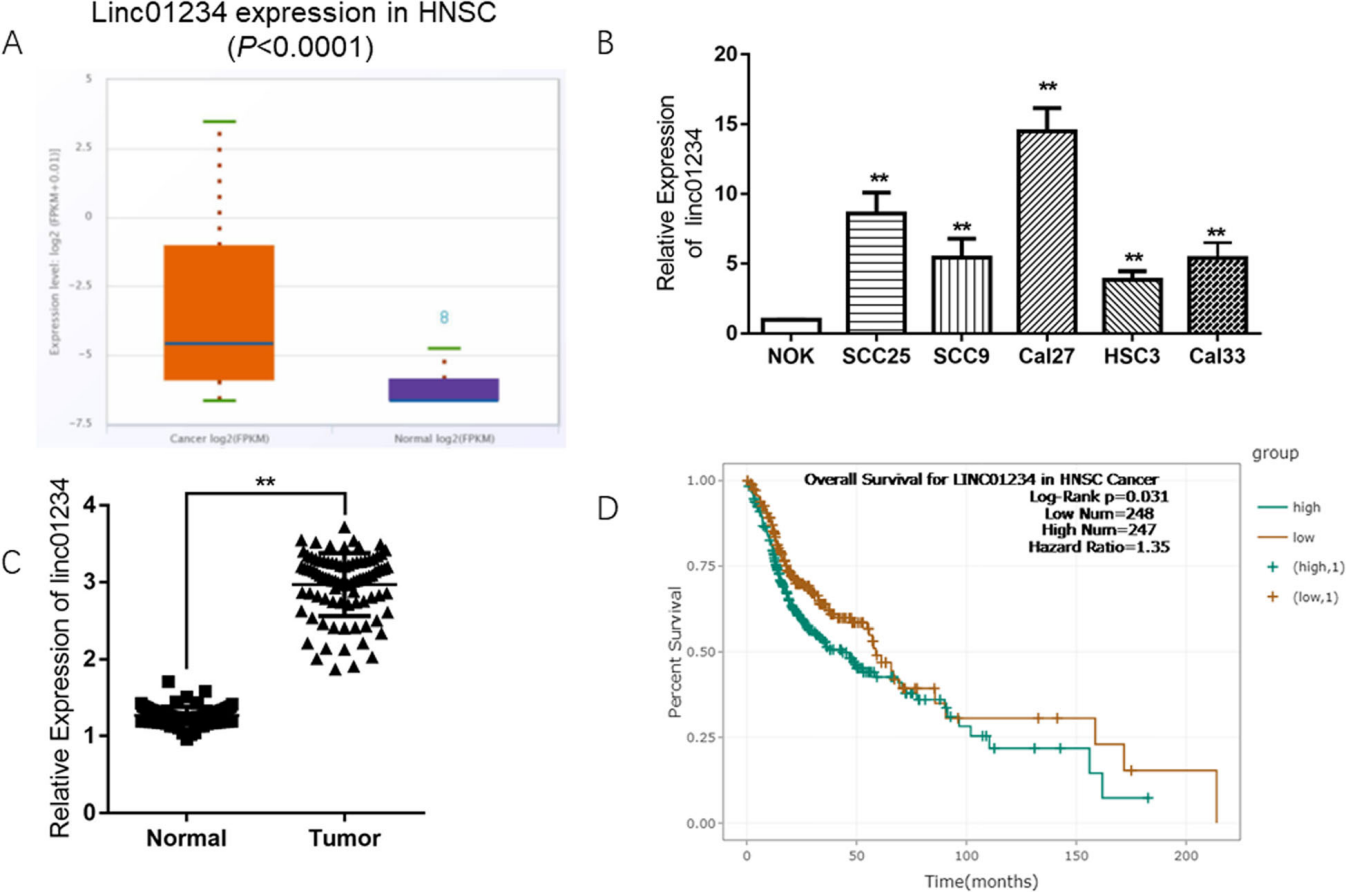
Linc01234 was upregulated in OSCC tissues and cell lines. **a** Linc01234 expression was significantly increased in OSCC tissues, compared with in adjacent non-tumor tissues via StarBase analysis. **b** Linc01234 expression was examined in OSCC and NOK cells via RT-qPCR assays. **c** Linc01234 expression was significantly increased in OSCC tissues, compared with in adjacent non-tumor tissues analyzed by RT-qPCR. **d** Kaplan-Meier analysis for the effects of Linc01234 expression on the OS of HNSC patients based on StarBase database. **,*P* < 0.01;***,*P* < 0.001 vs control

To explore the expression value and clinical significance of Linc01234 in OSCC, we first examined the levels of Linc01234 in the starBase database and specimens collected at our hospitals. As shown in ure 1A, Linc01234 was dramatically upregulated in HNSC samples (Cancer) compared with normal tissues (Normal) (*P* < 0.0001). We further detected the expression levels of Linc01234 in 88 OSCC specimens and adjacent oral normal tissues, which were collected from surgical resection at Affiliated Haikou Hospital and Xiangya Hospital. The RT-qPCR results indicated that the mRNA levels of Linc01234 in OSCC tissues were significantly higher than those in adjacent nontumor tissues (*P* < 0.01; Fig. 1c). Following culture of four OSCC cell lines and the negaFigtive control NOK cells, Linc01234 expression was found to be significantly increased in all OSCC cell lines (*P* < 0.05; Fig. 1b), compared with the NOK cell line.

### Linc01234 is closely associated with OSCC patient prognosis

To investigate the clinical significance of Linc01234 in OSCC progression, the association between Linc01234 expression and clinicopathological features was analyzed by the χ2 test. We found that Linc01234 expression was closely correlated with T stage, N stage and pathological stage (*P* < 0.05), however there was no statistical significance between Linc01234 and age, gender, tumor site or distant metastasis (Table 1). In addition, Cox analysis shown that Linc01234 expression could be an independent predictor of OSCC patient prognosis as well as T stage, N stage and advanced pathological stage (Table 2), indicating that Linc01234 could be an independent prognostic factor for OSCC with an aggressive phenotype. In addition, the starBase database analysis shown that high expression of Linc01234 in head and neck cancer patients was closely correlated with short overall survival (OS) (Fig. 1d).

**Table 1:** The association between linc01234 expression and clinicopathological parameters in patients with OSCC

Clinicopathological parameters	Linc01234 expression		χ2	*P* value
	Low(*N* = 44)	High(*N* = 44)		
Age			0.16	0.665
< 60	27	25		
≥ 60	17	19		
Gender			0.05	0.829
Male	25	26		
Female	19	18		
Tumor site			0.18	0.669
Tongue	22	24		
Non-tongue	22	20		
T stage			9.08	0.002
T_1–2_	32	18		
T_3–4_	12	26		
Lymphnode metastasis			3.93	0.048
No	32	23		
Yes	12	21		
Distance metastasis			0.72	0.395
Yes	6	9		
No	38	35		
Differentiation				
Well and moderately	34	26	4.87	0.027
Poorly	8	18

**Table 2:** Univariate and multivariate Cox proportional hazards analysis of linc01234 expression and OS in patients with OSCC

Clinicopathological parameters	Univariate Cox analysis		Multivariate Cox analysis	
	RR (95% CI)	*P* value	RR (95% CI)	*P* value
Age	0.91 (0.43–1.77)	0.588		
Gender	1.04 (0.65–1.51)	0.903		
Tumor site	1.15 (0.98–1.35)	0.094		
T stage	1.72 (1.18–2.26)	0.020	1.82 (1.42–2.94)	0.003
Lymphnode metastasis	1.41 (1.11–1.92)	0.036	1.52 (1.05–1.86)	0.031
Distance metastasis	0.95 (0.61–1.47)	0.834		
Differentiation	1.44 (1.18–1.82)	0.010	1.15 (1.02–1.30)	0.028
Linc01234 expression	1.64 (1.38–2.55)	0.008	1.99 (1.33–3.05)	0.001

### Silencing Linc01234 represses OSCC cell growth

**Fig. 2:**
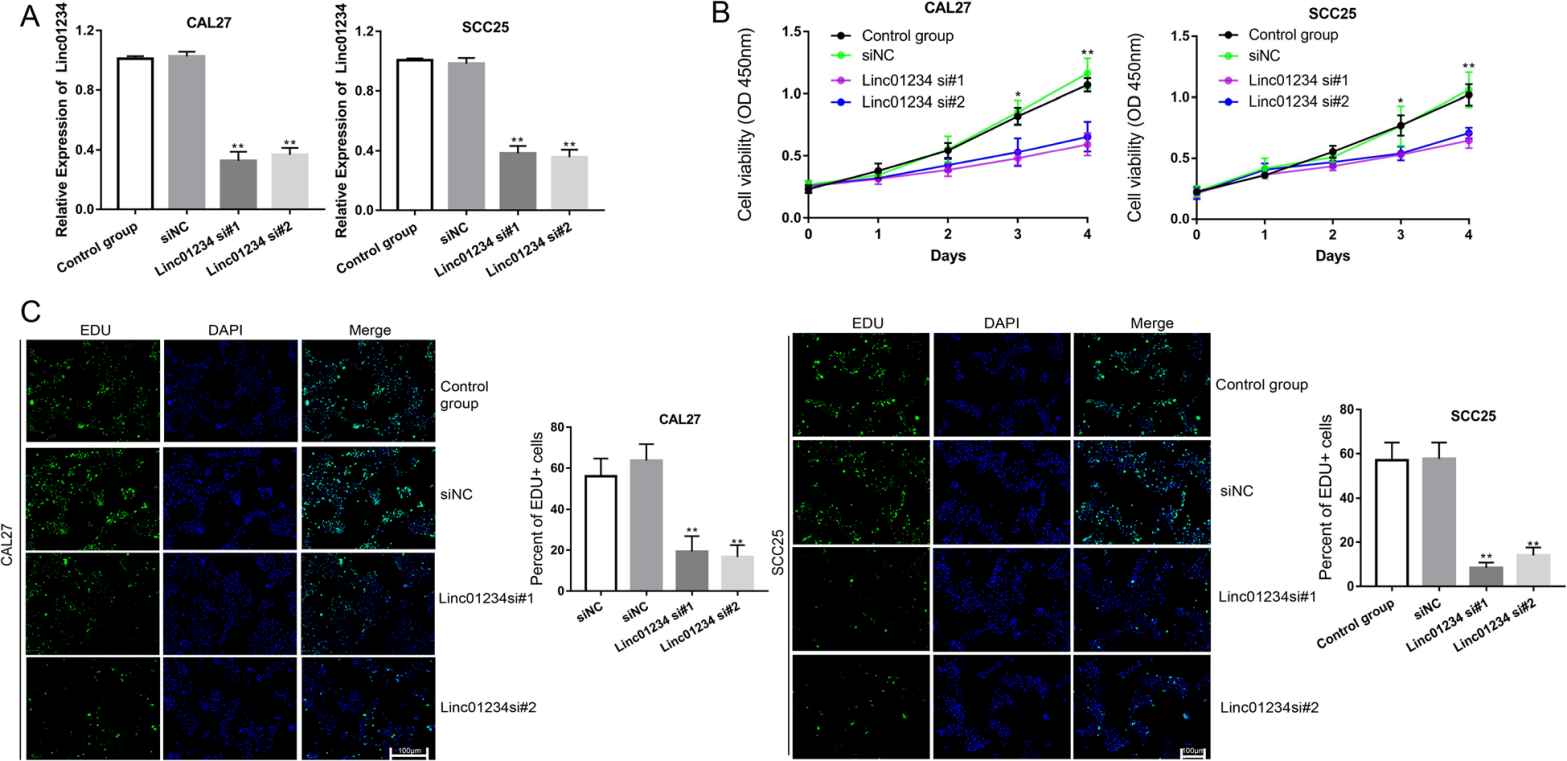
Decreased Linc01234 inhibits cell proliferation in vitro. **a** The relative expression of Linc01234 was detected with RT-qPCR when CAL27 and SCC25 cells were transfected with siNC, Linc01234 si#1 or Linc01234 si#2. **b** CCK8 assays were performed to detect the cell proliferation ability after CAL27 and SCC25 cells transfected with siNC, Linc01234 si#1 or Linc01234 si#2. **c** EDU assays were performed to detect the cell proliferation ability in CAL27 and SCC25 cells transfected with siNC, Linc01234 si#1 or Linc01234 si#2. *P < 0.05; **,*P* < 0.01 vs control

To explore the biological role of Linc01234 in OSCC, we transfected siRNAs targeting Linc01234 in CAL27 and SCC25 cells and examined Linc01234 levels by RT-qPCR. Following transfection, the siRNAs clearly decreased the transcription of Linc01234 in CAL27 and SCC25 cells (Fig. 2a), suggesting a high knockdown efficiency of these siRNAs. Using CCK8 assays, the proliferative rates were shown to be significantly decreased by Linc01234 inhibition in CAL27 cells and SCC25 cells (Fig. 2b). Furthermore, Edu-positive cells among Linc01234 knockdown OSCC cells were remarkedly lower than those in the control group and siNC group (Fig. 2c), indicating a decreased DNA synthesis ability in OSCC cells with Linc01234 siRNAs. Our data suggested that Linc01234 promotes OSCC cell proliferation by enhancing DNA synthesis.

### Linc01234 knockdown inhibits migration and invasion in OSCC cells

**Fig. 3:**
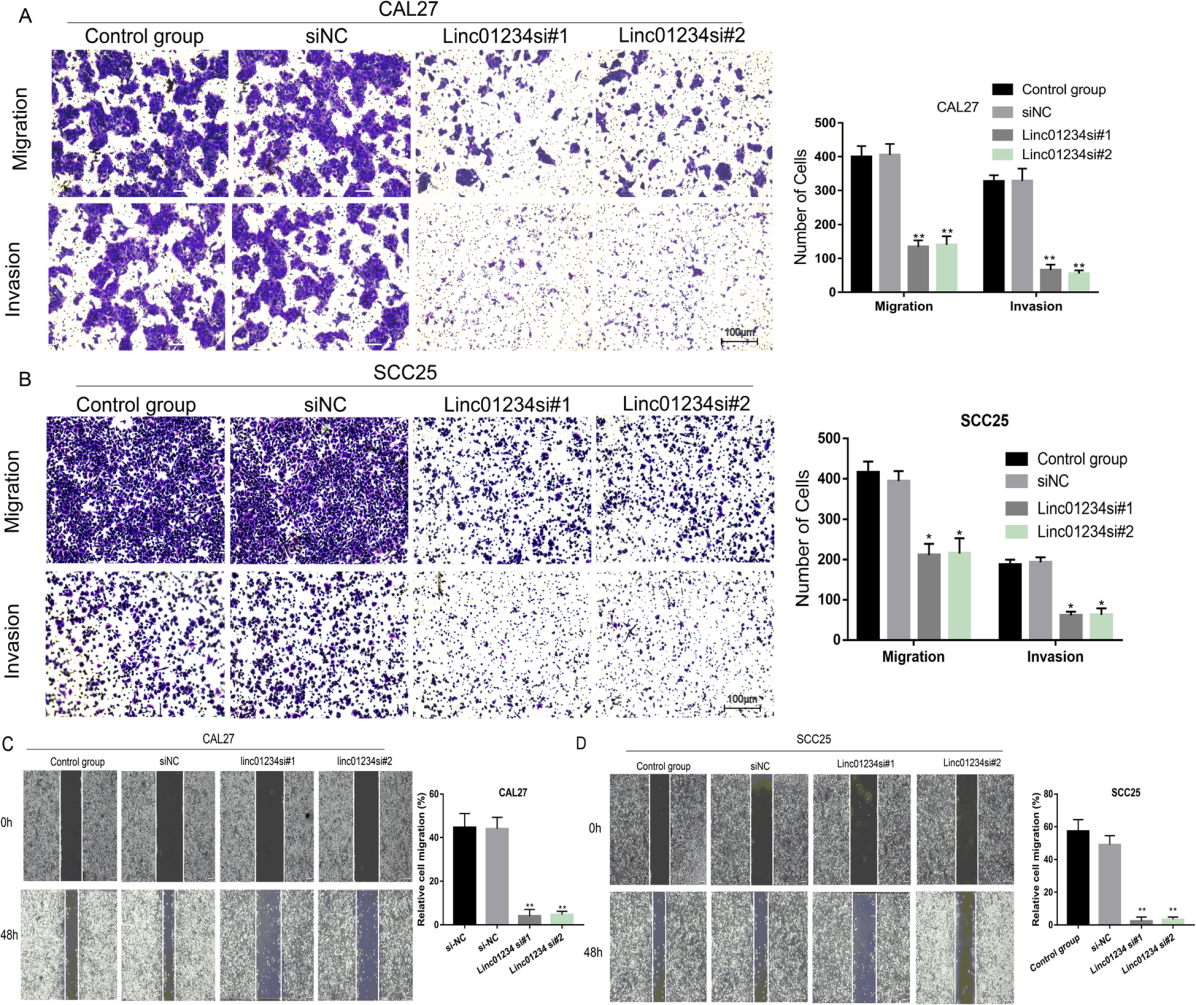
Decreased Linc01234 inhibits migration and invasion in vitro. **a**, **b** The ability of cell migration and invasion in CAL27 and SCC25 cells with Linc01234 knockdown was detected by Transwell assays. **c**, **d** The migration ability of CAL27 and SCC25 cells with Linc01234 knockdown was detected by wound healing assays. * *P* < 0.05; **,*P* < 0.01 vs control

In view of the strong association of Linc01234 expression with lymph node metastasis (N stage), the effect of Linc01234 on cell migration and invasion in OSCC cells was further explored. As shown in Fig. 3a and b, a large number of CAL27 and SCC25 cells in the control and siNC groups migrated to the lower surface of the upper chamber. However, a smaller number of OSCC cells migrated to the lower surface in the Linc01234 knockdown groups. Figure 3c and d indicate that the wound healing area of the control and siNC groups was significantly greater than that in the Linc01234 knockdown group. These observations indicated that Linc01234 may function as a positive regulator of cell metastasis in OSCC.

### Linc01234 sponges miR-433-3p in OSCC cells

**Fig. 4:**
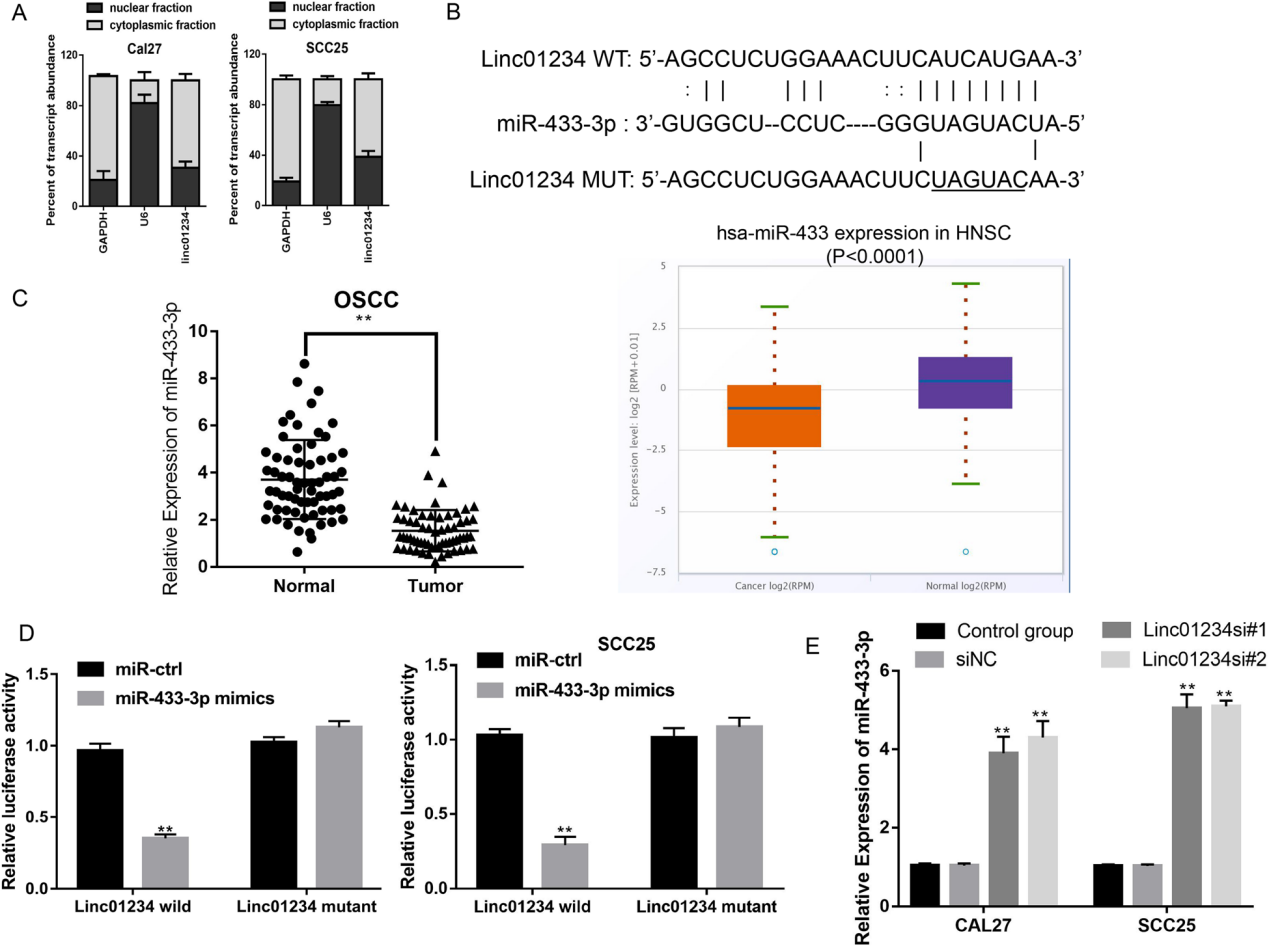
Linc01234 could sponge miR-433-3p in OSCC. **a** The cellular localization of Linc01234 was determined by Subcellular fractionation and RT-qPCR assay. GAPDH: cytoplasmic control, U6: nuclear control. **b** Schematic illustration of the predicted binding sites between Linc01234 and miR-433-3p and mutation of potential miR-433-3p binding sequence in Linc01234. **c** miR-433-3p expression was examined in OSCC tissues using RT-qPCR and StarBase analysis. **d** Luciferase reporter assay indicated the direct bind between Linc01234 and miR-433-3p. **e** The mRNA level of miR-433-3p after Linc01234 knockdown was observed by RT-qPCR assay. *P < 0.05; **,*P* < 0.01 vs control

Numerous studies have reported that lncRNAs can act as microRNA (miRNA) sponges, to regulate the biding of endogenous miRNAs to target mRNAs and inhibit the expression of the target mRNAs [16]. First, we found that Linc01234 was mainly expressed in the cytoplasm of CAL27 and SCC25 cells by RT-qPCR (Fig. 4a). Then, we screened the potential target miRNAs using LncBase Predicted version (v.)2 of DIANA tools and obtained 35 potential miRNAs. Screening the Pubmed database, we determined that miR-433-3p, a well-known tumor suppresser, was a possible downstream target of Linc01234 (Fig. 4b). Furthermore, miR-433-3p was decreased in OSCC samples compared with normal tissues, as analyzed by RT-qPCR and the starBase database (Fig. 4c). Then, we performed a dual-luciferase assay to validate this hypothesis. Our results indicated that the miR-433-3p mimics + Linc01234 wild-type (Linc01234 wild) group but not the miR-433-3p mimics + Linc01234 mutant type (Linc01234 mutant) group shown obviously reduced luciferase activity in OSCC cells (Fig. 4d). Additionally, Linc01234 inhibition significantly increased miR-433-3p expression levels in CAL27 and SCC25 cells (Fig. 4e). In summary, we confirmed that Linc01234 could sponge miR-433-3p in OSCC cells.

### miR-433-3p directly binds to the 3′UTR of PAK4

**Fig. 5:**
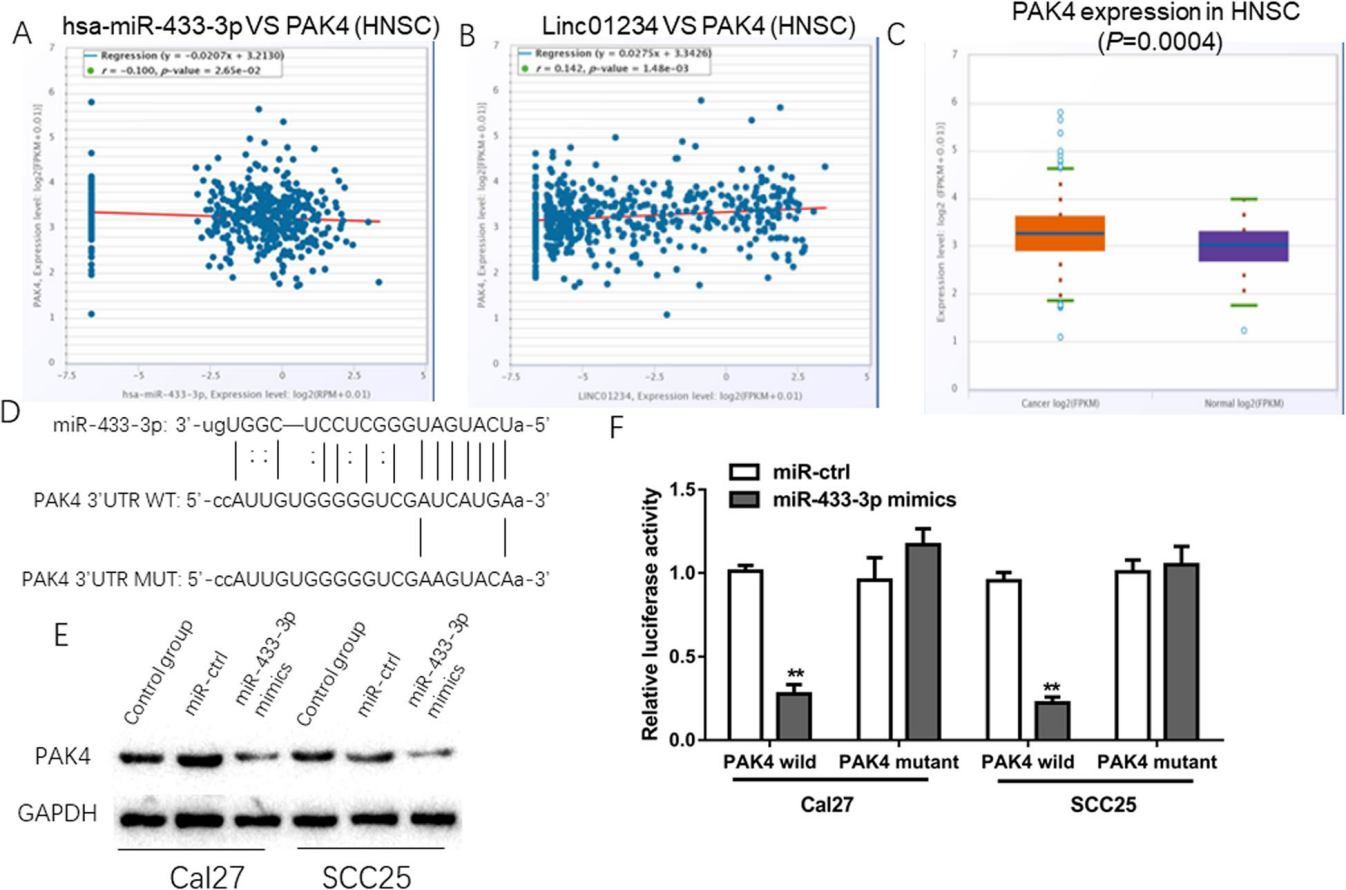
PAK4 is a target gene of miR-433-3p in OSCC. **a and b** revealed the correlation between PAK4 expression and miR-433-3p expression or Linc01234 expression in HNSC tissues. **c** PAK4 expression in HNSC tissues and normal tissues was veritied by StarBase analysis. **d** The putative binding sites of miR-433-3p and PAK4 was shown. **e** The expression of PAK4 was detected by Western Blot in OSCC cells with miR-433-3p overexpression. **f** The direct interaction between miR-433-3p and PAK4 in OSCC cells was examined by luciferase reporter assay. *P < 0.05; **,*P* < 0.01 vs control

We confirmed that Linc01234 served as an oncogene in OSCC progression and demonstrated that Linc01234 functioned as a ceRNA regulating miR-433-3p expression. Thus, we further investigated the potential target of the Linc01234/miR-433-3p axis. Using the TargetScan and starBase tools, we found that the expression of PAK4 was positively correlated with Linc01234, but negatively correlated with miR-433-3p, and miR-433-3p have binding sites in the 3′UTR of PAK4. In addition, PAK4 was remarkedly downregulated in head and neck cancer analyzed by starBase (Fig. 5a-d). Moreover, we compared the endogenous expression of miR-433-3p and PAK4 in different cell lines. The RT-qPCR and WB results indicated an opposite expression pattern between miR-433 and PAK4 in the different OSCC cells (Additional file 2: Figure S2). To confirm whether PAK4 was a direct target of miR-433-3p, we purchased wild-type and mutant luciferase reporter plasmids containing the complementary sequence of miR-433-3p in PAK4 and cotransfected miR-433-3p mimics and these luciferase reporter vectors into OSCC cells. The results indicated that the luciferase activities dramatically declined in OSCC cells cotransfected with miR-433-3p mimics and the wild-type PAK4 vector, but no change in luciferase activity of OSCC cells transfected with the mutant PAK4 plasmid (Fig. 5f). Furthermore, PAK4 expression was significantly suppressed by miR-433-3p overexpression or Linc01234 inhibition in CAL27 and SCC25 cells (Fig. 5e, Additional file 1: Figure S1). PAK4 knockdown also obviously repressed the migration and invasion of OSCC cells (Additional file 3: Figure S3). Therefore, we propose that PAK4 is a direct target of miR-433-3p in OSCC cells.

## Discussion

Recently, increasing evidence has demonstrated that an aberrant lncRNA could facilitate the development and progression of solid tumors and particular lncRNA was identified as an independent biomarker [17–19]. Due to the unknown biological functions of lncRNAs and limited understanding of the molecular mechanisms, more studies are needed. Our present study investigated the OSCC prognosis-related lncRNA---Linc01234, and elucidated its functional roles in OSCC progression.

In the present research, we found that Linc01234 was significantly upregulated in clinical OSCC samples and cell lines. High Linc01234 expression was positively related to advanced T stage, lymph node metastasis and poor pathological differentiation. In addition, OSCC patients with high Linc01234 expression had a worse overall survival (OS) than patients with low Linc01234 expression based on database analysis. Cox analysis also indicated that Linc01234 expression could be an independent predictor of OSCC patient prognosis as well as T stage, N stage and advanced pathological stage. In biological functional experiments, Linc01234 inhibition prominently contributed to the decreased proliferative activity and metastasis of CAL27 and SCC25 cells. In summary, our results suggested that Linc01234 plays a cancer-promoting role in cell growth and metastasis in OSCC.

Growing evidence indicates that lncRNAs and mRNAs can cross-regulate each other by competing for shared miRNA response elements (MREs) [16, 20]. Specifically, many lncRNAs act as sponges in the regulation of miRNA target genes involved in OSCC carcinogenesis [21, 22]. miR-433, a well-characterized miRNA, was found to be a tumor suppressor in different neoplasms [23, 24]. Furthermore, Wang et al. reported that miR-433 was downregulated in OSCC tissues and demonstrated that miR-433 expression markedly suppressed cell proliferation, invasion and migration by targeting HDAC6 [25]. In our study, we found that Linc01234 contained miRNA response elements for miR-433-3p with 13 nt complementary sequence. Dual-luciferase assays confirmed a direct correlation between miR-433-3p and Linc01234. RT-qPCR results shown that Linc01234 silencing increased the expression levels of miR-433-3p, which was dramatically downregulated in head and neck cancer tissues. Our data suggested that Linc01234 may act as a ceRNA (competing endogenous RNA) of miR-433-3p to relieve the repressive effect of miR-433-3p on its target. However, the underlying mechanism of the Linc01234/miR-433-3p axis in OSCC remains unclear.

p21-Activated kinase 4 (PAK4), a member of the PAK family, regulates a wide range of cellular functions, including cell adhesion, migration, proliferation, and survival [26, 27]. Previous studies have reported that dysregulation of PAK4 expression contributes to the development and progression of various tumors [28, 29]. Several studies have reported that PAK4 could be regulated by many miRNAs in various cancers, including miR-485 and miR-199a-3p [30–32]. In OSCC, PAK4 serves as a super enhancer-associated candidate oncogene and promotes the proliferation of OSCC cells [33]. In our study, we identified PAK4 as a potential target of miR-433-3p based on TargetScan and starBase prediction analyses. Dual-luciferase assays confirmed that miR-433-3p could bind to PAK4 directly. Furthermore, PAK4 protein levels in CAL27 and SCC25 cells with overexpressing miR-433-3p were significantly inhibited. Overall, our findings suggest that Linc01234 modulates OSCC carcinogenesis through miR-433-3p-regulated PAK4.

## Conclusion

In summary, our study confirmed that Linc01234 promotes OSCC progression through the Linc01234/miR-433-3p/PAK4 axis and may serve as a new diagnostic marker or target for the treatment of OSCC patients.

## Supplementary information

**Additional file 1: Figure S1.** Linc01234 knockdown inhibits PAK4 expression. The relative expression of PAK4 was detected with RT-qPCR(A) and Western Blot(B) when CAL27 and SCC25 cells were transfected with siNC, Linc01234 si#1 or Linc01234 si#2. *P < 0.05; **,*P* < 0.01 vs control.

**Additional file 2: Figure S2.** The endogenous expression of miR-433-3p and PAK4 in different cell lines. (A) The endogenous expression of miR-433-3p was examined in OSCC and NOK cells via RT-qPCR assays. (B and C) The expression of PAK4 was examined in OSCC and NOK cells via RT-qPCR and Western Blot. *P < 0.05; **,*P* < 0.01 vs control.

**Additional file 3: Figure S3.** PAK4 knockdown inhibits migration and invasion in OSCC cells. The relative expression of PAK4 was detected with RT-qPCR(A) and Western Blot(B) when CAL27 and SCC25 cells were transfected with siNC, PAK4 si#1 or PAK4 si#2. (C) The ability of cell migration and invasion in CAL27 and SCC25 cells with PAK4 knockdown was detected by Transwell assays. *P < 0.05; **,*P* < 0.01 vs control.

**Additional file 4: Figure S4.** is the result of mycoplasma contamination detection.

## Data Availability

The datasets used and/or analyzed during the current study are available from the corresponding author on request.

## Abbreviations

ceRNA: Competitive endogenous RNA
HDAC6: Histone Deacetylase 6
LncRNA: Long non-coding RNA
MRE: mirna response element
NOK: Normal oral keratinocytes
OS: Overall survival
OSCC: Oral squamous cell carcinoma
PAK4: P21-Activated kinase 4
