# Supplementary figures and images for: Linc01234 promotes cell proliferation and metastasis in oral squamous cell carcinoma via miR-433/PAK4 axis
Source: BMC Cancer. 2020 Feb 10;20:107. doi: 10.1186/s12885-020-6541-0 (PMC7011552; doi:10.1186/s12885-020-6541-0)

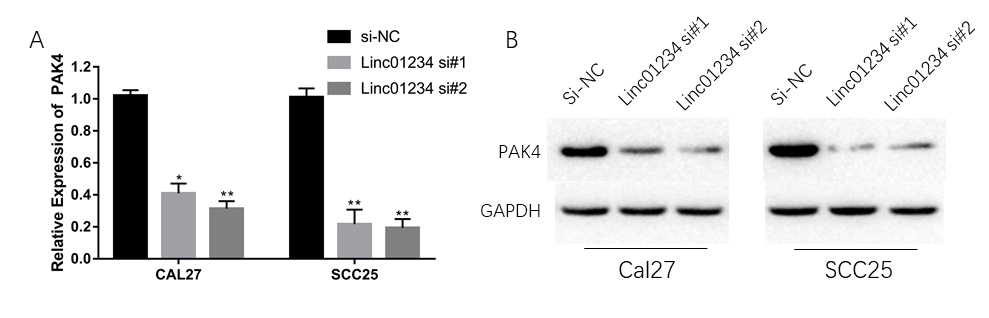

Supplement: Supplementary file 1 — Additional file 1: Figure S1. Linc01234 knockdown inhibits PAK4 expression. The relative expression of PAK4 was detected with RT-qPCR(A) and Western Blot(B) when CAL27 and SCC25 cells were transfected with siNC, Linc01234 si#1 or Linc01234 si#2. *P < 0.05; **,P < 0.01 vs control. [file 12885_2020_6541_MOESM1_ESM.tif]

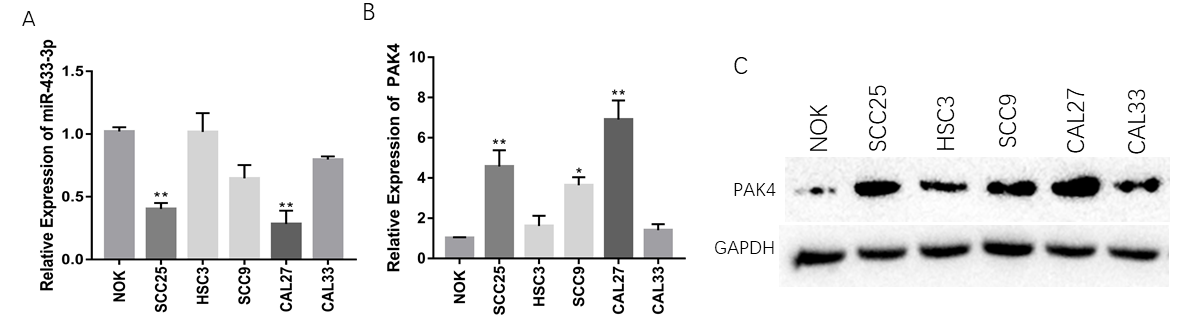

Supplement: Supplementary file 2 — Additional file 2: Figure S2. The endogenous expression of miR-433-3p and PAK4 in different cell lines. (A) The endogenous expression of miR-433-3p was examined in OSCC and NOK cells via RT-qPCR assays. (B and C) The expression of PAK4 was examined in OSCC and NOK cells via RT-qPCR and Western Blot. *P < 0.05; **,P < 0.01 vs control. [file 12885_2020_6541_MOESM2_ESM.tif]

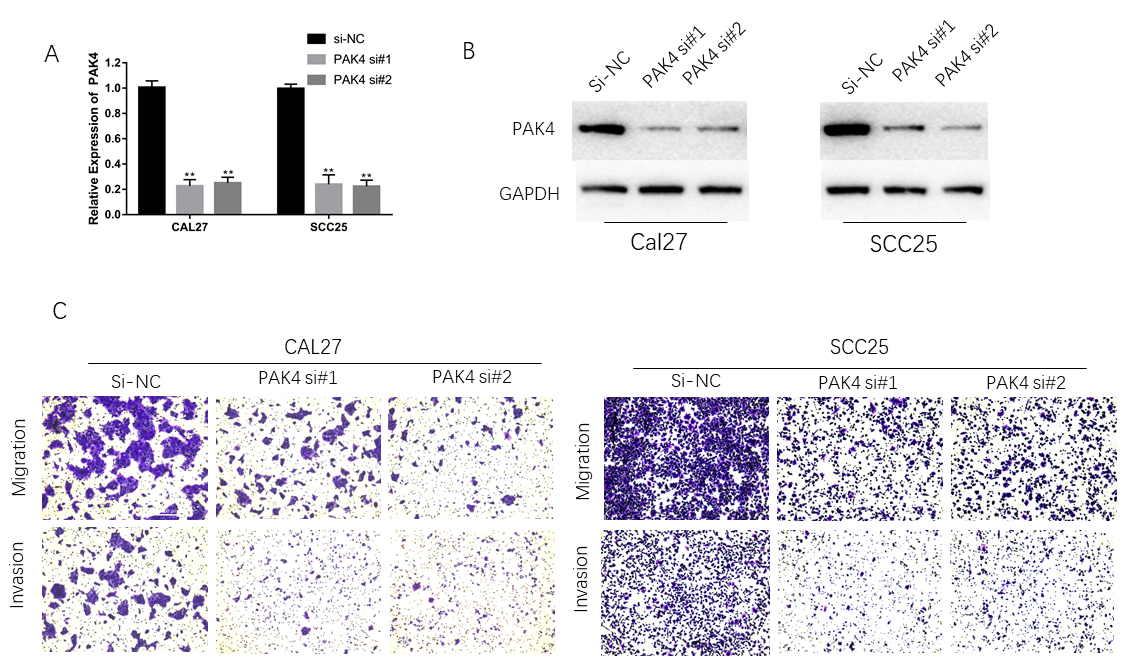

Supplement: Supplementary file 3 — Additional file 3: Figure S3. PAK4 knockdown inhibits migration and invasion in OSCC cells. The relative expression of PAK4 was detected with RT-qPCR(A) and Western Blot(B) when CAL27 and SCC25 cells were transfected with siNC, PAK4 si#1 or PAK4 si#2. (C) The ability of cell migration and invasion in CAL27 and SCC25 cells with PAK4 knockdown was detected by Transwell assays. *P < 0.05; **,P < 0.01 vs control. [file 12885_2020_6541_MOESM3_ESM.tif]

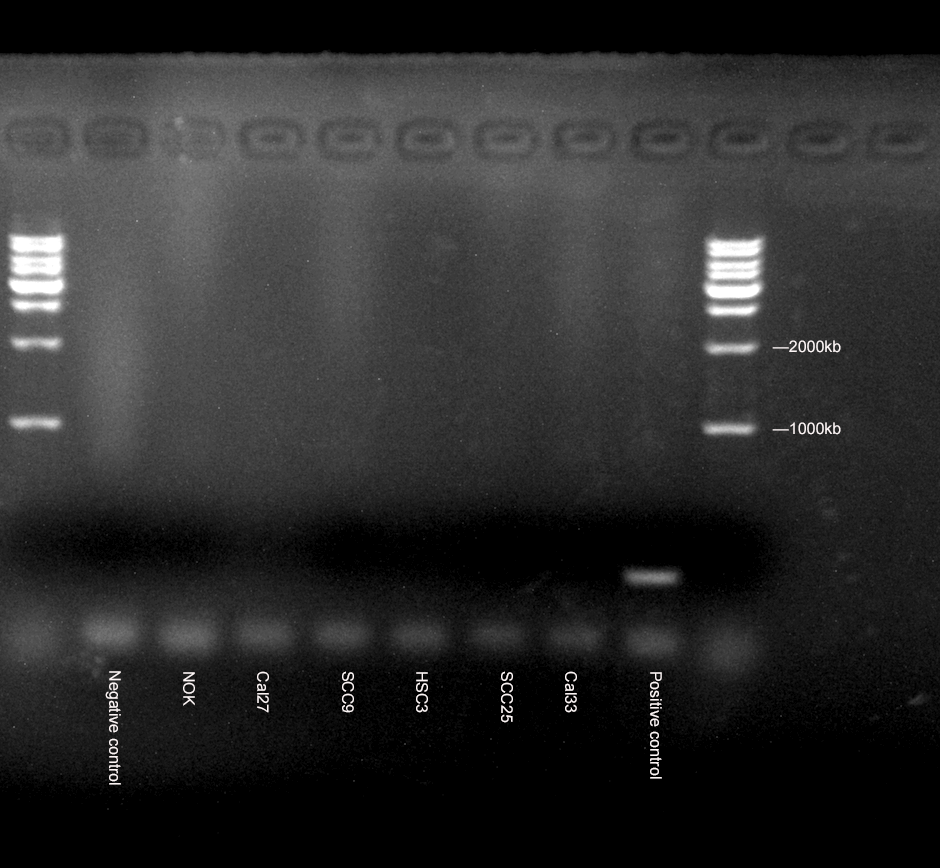

Supplement: Supplementary file 4 — Additional file 4: Figure S4. is the result of mycoplasma contamination detection. [file 12885_2020_6541_MOESM4_ESM.tif]
